# Aligning antimicrobial resistance surveillance with schistosomiasis research: an interlinked One Health approach

**DOI:** 10.1093/trstmh/trae035

**Published:** 2024-06-06

**Authors:** Angus M O'Ferrall, Janelisa Musaya, J Russell Stothard, Adam P Roberts

**Affiliations:** Department of Tropical Disease Biology, Liverpool School of Tropical Medicine, Pembroke Place, Liverpool L3 5QA, UK; Malawi Liverpool Wellcome Clinical Research Programme, Queen Elizabeth Central Hospital, College of Medicine, P.O. Box 30096 Chichiri, Blantyre 3, Malawi; Department of Tropical Disease Biology, Liverpool School of Tropical Medicine, Pembroke Place, Liverpool L3 5QA, UK; Department of Tropical Disease Biology, Liverpool School of Tropical Medicine, Pembroke Place, Liverpool L3 5QA, UK

**Keywords:** AMR, Enterobacterales, ESBL, freshwater, Malawi, snail-borne diseases

## Abstract

One Health surveillance involves the analysis of human, animal and environmental samples, recognising their interconnectedness in health systems. Such considerations are crucial to investigate the transmission of many pathogens, including drug-resistant bacteria and parasites. The highest rates of antimicrobial resistance (AMR)-associated deaths are observed in sub-Saharan Africa, where concurrently the waterborne parasitic disease schistosomiasis can be highly endemic in both humans and animals. Although there is growing acknowledgment of significant interactions between bacteria and parasites, knowledge of relationships between schistosomes, microbes and AMR remains inadequate. In addition, newly emergent research has revealed the previously underappreciated roles of animals and the environment in both AMR and schistosomiasis transmission. We consider shared environmental drivers and colonisation linkage in this narrative review, with a focus on extended-spectrum beta-lactamase-mediated resistance among bacteria from the Enterobacteriaceae family, which is exceedingly prevalent and responsible for a high burden of AMR-associated deaths. Then we examine novel findings from Malawi, where the landscapes of AMR and schistosomiasis are rapidly evolving, and make comparisons to other geographic areas with similar co-infection epidemiology. We identify several knowledge gaps that could be addressed in future research, including the need to characterise the impact of intestinal schistosomiasis and freshwater contact on intestinal AMR colonisation, before proposing a rationale for connecting AMR surveillance and schistosomiasis research within a One Health framework.

## Introduction

One Health is an integrated approach to understanding and addressing health issues that recognises the interdependence of humans, animals and the environment.^1^ The majority of emerging infectious diseases reported globally come from animals.^1^ Broader One Health considerations are equally important when considering the control of infectious diseases with more ancient origins. The acquisition of environmental (e.g. water), human and animal (e.g. faecal) samples can facilitate parallel surveillance of many pathogens using field-based and/or molecular biology diagnostic methodologies. Multiple use of samples can, in theory, reveal important information on the linkage of transmission and co-infection dynamics for diseases caused by various pathogens, including bacteria and parasites. To illustrate the possibilities of this approach, we explore the potential linkage between antimicrobial resistance (AMR) in bacteria and an important waterborne parasitic disease.

Many neglected tropical diseases (NTDs) are caused by metazoan parasites, which are generally most prevalent in low- and middle-income countries (LMICs). NTDs are inextricably linked with animal health and production systems alongside closely tied environmental factors.^2^ Schistosomiasis is an example of one such NTD—a waterborne helminth infection caused by flatworm blood flukes of the genus *Schistosoma*, the life cycles of which are wholly dependent on intermediate snail hosts in freshwater environments.^3^ The disease, of which there are two types (urogenital and intestinal), is conservatively estimated to affect >230 million people globally^4^ and is responsible for significant morbidity and mortality.^5^ In sub-Saharan Africa (SSA), where an estimated 90% of schistosomiasis-infected people live,^5^ urogenital disease is predominantly caused by *Schistosoma haematobium* and intestinal disease by *Schistosoma mansoni*.^3^ Further, other *Schistosoma* species infect a wide range of mammals.^6^ Common to all schistosomes is the percutaneous route of infection and ensuing chronic disease after schistosome eggs are deposited within host tissues.^4^ Contamination of freshwater via urination and defecation perpetuates transmission cycles, as eggs that are shed from the body go on to (re)infect intermediate host snails.^3^ Schistosomiasis is therefore especially prevalent in communities where contact with infested water is common. Malacologic (snail) control and improvement of water, sanitation and hygiene (WASH) infrastructures are crucial One Health control measures to improve human and animal health in areas where schistosomiasis is endemic.^7^

One Health approaches are also emerging as key considerations in tackling the growing threat to global public health and biosecurity posed by AMR, and it may be possible to capitalise on both surveillance and research between interlinked transmission routes. AMR is a natural evolutionary phenomenon resulting from selection pressures introduced by large-scale antimicrobial use.^8^ In 2019, bacterial AMR was associated with an estimated 4.95 million deaths globally.^9^ The highest rates of all-age deaths are observed in LMICs.^9^ The annual mortality rate per 100 000 persons is predicted to be 98.9 in SSA,^9^ where comprehensive surveillance data are lacking, the quality of available antibiotics is often poor and limited antibiotic access restricts prescription choices.^10^ While improving pharmaceutical systems is fundamental in managing AMR,^11^ strategies to marshal the public health threat posed by AMR must extend beyond medical prescribing. The roles of animals and environmental contamination in complex AMR transmission pathways are in focus now more than ever, with multidrug-resistant, disease-causing bacteria spreading across various environmental sources, including freshwater.^12–14^ Considering this, the potential for simultaneous exposure to AMR bacteria and freshwater parasites is increasing in communities lacking adequate WASH infrastructure, where environmental water bodies, such as Lake Malawi, are particular strongholds for schistosomiasis transmission.

Bacteria and parasites share complex and intricate relationships, the investigation and exploitation of which is a new focus in global health research.^15^ While schistosomes themselves can be colonised with bacteria,^16^ schistosomiasis is also associated with constitutional changes to the host intestinal microbiome,^17^ which may in turn affect the host resistome.^18^ In this narrative review, we consider and assess the relationships between AMR and schistosomiasis through a One Health lens. Our perspective is to identify knowledge gaps and highlight avenues for the discovery of previously unrecognised AMR drivers in LMICs. To accomplish this objective, comprehensive searches of published and preprint literature were carried out to identify studies investigating two or more of the following topics: AMR, schistosomiasis, freshwater or wastewater and One Health or environmental transmission.

## The importance of AMR in Enterobacteriaceae

A key feature in the evolution and spread of AMR is the exchange of mobile genetic elements between bacteria, occurring by horizontal gene transfer, a phenomenon that is exemplified by the global spread of extended-spectrum beta-lactamase (ESBL)-mediated resistance in Enterobacteriaceae.^19^ The Enterobacteriaceae family, of the order Enterobacterales, includes Gram-negative bacteria that make up part of the normal intestinal flora as well as those that are often pathogenic.^20^ Examples of clinically relevant Enterobacteriaceae include *Escherichia coli* and *Klebsiella pneumoniae*, which rank first and third in the list of pathogens associated with deaths due to bacterial AMR worldwide.^9^ Third-generation cephalosporin-resistant Enterobacterales are often overlooked in the development of new antimicrobial agents^21^ despite being listed by the World Health Organization as critical priority pathogens.^22^ With broad-spectrum treatment cover, cost-effectiveness and simple dosing regimens, third-generation cephalosporins, particularly ceftriaxone, are frequently prescribed in the hospital setting in SSA.^23–25^ In Malawi, the rising rate of third-generation cephalosporin resistance observed over the last 2 decades in Enterobacteriaceae^26^ is now significantly associated with increased mortality and length of hospital admission in patients with bloodstream infections.^27^

## Shared environmental transmission drivers

The transmission of antimicrobial resistance genes (ARGs) across bacterial populations is complex and multifactorial, and investigation of the role of environmental contamination in spreading resistance among Enterobacteriaceae is an increasingly recognised area of research. Hospitals are an obvious source of drug resistance given the high rates of antibiotic prescribing and administration; of note, multidrug-resistant Enterobacteriaceae are prevalent in hospital sewage and wastewater.^28–30^ Subsequent environmental contamination and the need for improved environmental control measures are exemplified by the persistence of ESBL genes in aquatic habitats^31^ and in soil and vegetables following water or manure exposure.^32^ To this end, parallels can be drawn between strategies for the control of AMR transmission and the control of waterborne zoonotic helminthiases such as schistosomiasis in LMICs, where improved WASH infrastructures can play a crucial role in breaking transmission cycles but are often poorly implemented.^7^

**Figure 1.**
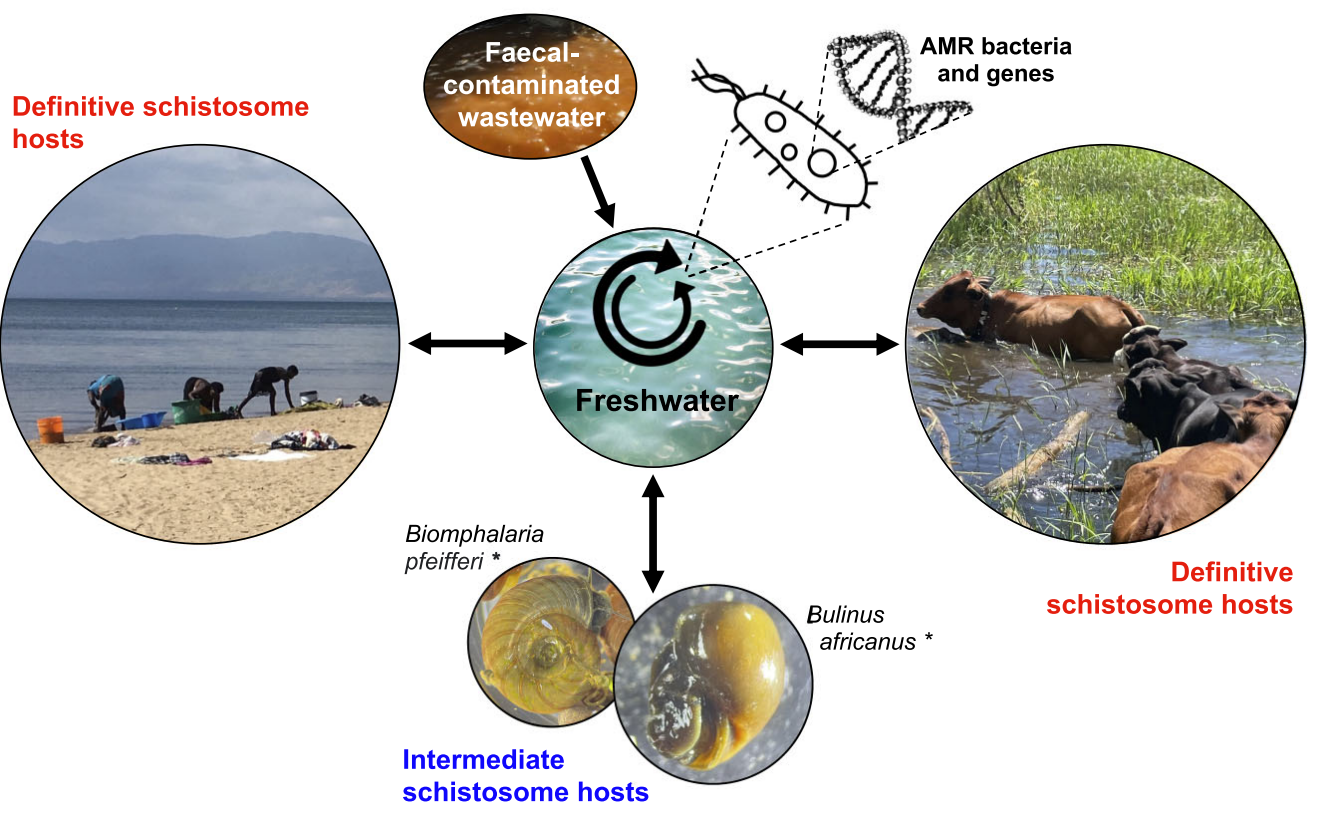
Potential transmission of AMR bacteria and genes between the environment and schistosome hosts (pictured: [left] humans, [right] cattle, [bottom] freshwater snails). Arrows indicate directionality of AMR transfer. *Other freshwater snail species (not pictured) can also transmit schistosomiasis.

Despite gut colonisation with AMR bacteria being common in definitive schistosome hosts, with numerous freshwater environments being polluted with various excreta in schistosomiasis-endemic areas, connections between AMR and schistosomes are poorly recognised at present. This extends to environmental factors that influence the transmission of schistosomiasis, and potentially also AMR. For instance, the roles of aquatic invertebrates should be considered, such as freshwater snails that transmit schistosomiasis, which may also ingest and filter various bacteria in the environment. To our knowledge, no appraisal of AMR colonisation in freshwater snails has taken place in Africa. While this knowledge gap extends globally in terms of genomic AMR surveillance, phenotypic susceptibility testing has demonstrated resistance to a range beta-lactam antibiotics in Enterobacteriaceae isolated from the microbiota of *Biomphalaria glabrata* snails in Brazil, which act as an intermediate host for *S. mansoni*.^33^ Intermediate snail hosts, which feed by grazing in their environments,^34,35^ may therefore be seen as potential AMR reservoirs and dispersers in schistosomiasis-endemic regions where poor WASH infrastructure is also associated with widespread freshwater contamination with ESBL-producing Enterobacterales.^14,36^ Considering this, freshwater environments may be a central source for advancing AMR in schistosomiasis-endemic areas where contamination with human and animal excreta is common (Figure 1).

## Associations between schistosomiasis, the gut microbiome and AMR

Broadly, knowledge of the effects that helminths have on the gut microbiome is beginning to emerge. Helminth infections are associated with potent immunomodulatory effects that are thought to play a key role in the development of metabolic and immune system development.^37^ In countries where helminthiases are endemic, gut microbiota often exhibit greater diversity.^38^ In fact, the immunosuppressive effects of helminth infections thought to be responsible for this are a potential factor in the lower observed incidence of autoinflammatory diseases such as inflammatory bowel disease in many LMICs.^39^ Yet questions remain, including whether all helminths interact with the intestinal bacteria in a similar fashion and whether interbacterial dynamics influence the establishment of helminth infections.^40^

In SSA, the majority of studies investigating the effects of schistosomiasis on the human intestinal microbiota report on urogenital infections caused by *S. haematobium*. Despite not being a gut-resident parasite, there is strong evidence from comparative studies that *S. haematobium* infection causes significant changes in the gut microbiome at multiple taxonomic levels.^41–43^ The differences noted between *S. haematobium*–infected and –uninfected groups indicate that infection is associated with intestinal dysbiosis,^42^ but the microbial signatures reported in people with urogenital schistosomiasis across these studies do not include changes in the abundance of Enterobacterales, which are of interest both clinically and for public health in the context of AMR.

Given that the pathophysiological processes described in intestinal schistosomiasis not only involve immunomodulation, but lead to structural enteropathies,^44^ intestinal infections may exert additional selective pressures associated with microbiome alterations. In mice, *S. mansoni* infection is associated with reduced microbial diversity within individuals (alpha-diversity) but is associated with increased beta-diversity—the microbial diversity observed between individuals.^45^ Mouse models also suggest that baseline microbial diversity affects susceptibility to *S. mansoni* infection.^46^ Assessment of associations between *S. mansoni* and the human intestinal microbiome remain less comprehensive, yet one study of 34 school-age children (SAC) and pre-school-age children (PSAC) in Côte d'Ivoire identified that bacteria from the genus *Klebsiella* were significantly more abundant in children infected with *S. mansoni*.^47^ This may enable greater transmission of ESBL genes, which are capable of spreading across a range of *K. pneumoniae* lineages.^48^

Microbiome changes in *Schistosoma japonicum* infection are dynamic during progression through the different stages of disease chronicity.^49^ In China, advanced *S. japonicum* infection in human hosts has been shown to increase the abundance of *Bacteroides* in the intestinal microbiome,^49,50^ the presence of which has been linked with reduced levels of ESBL-producing Enterobacteriaceae carriage in a study comparing intestinal microbiota composition in carriers and non-carriers in Thailand.^51^ Meanwhile, the effect of anthelmintic treatment with praziquantel on the gut microbiome appears to be insignificant, or at least is undetectable against the background changes caused by *Schistosoma* infection in the first instance.^41,47,52^ The above findings illustrate how relationships between schistosomes, microbes and AMR are complex, but whether the microbial selective pressures and constitutional microbiome changes associated with schistosome infections affect the abundance and transmission of ARGs within human and animal populations is yet to be fully understood.

To our knowledge, only one published study explored this novel line of research—a cross-sectional study of 113 PSAC in a Zimbabwean village investigating the effects of urogenital schistosomiasis on the faecal resistome (i.e. all ARGs within a sample).^43^ In the study, which analysed shotgun metagenomic sequencing outputs, no differences in the resistome between *S. haematobium*–infected PSAC compared with uninfected PSAC were detected. Alongside the limited sample size in one geographical setting, lack of effect detection in the aforementioned study does not rule out the possibility that schistosomiasis influences the resistome predominantly for the following reasons: the diversity of the gut microbiome is known to increase with age across locations, LMIC-inclusive,^53^ so resistome alterations may be more detectable in older age groups than in PSAC, and no studies have yet explored the impact of intestinal schistosomiasis or *Schistosoma* species co-infections on AMR.

## One Health case studies in SSA

As increasing schistosomiasis prevalence and widespread ESBL-producing Enterobacteriaceae colonisation have both recently been detected in Malawi,^14,54,55^ the landlocked country that is home to one of Africa's Great Lakes is a focus for increased monitoring of both health issues. *Schistosoma haematobium*, transmitted by *Bulinus* species freshwater snails, has been endemic in communities around Lake Malawi for many years, but the emergence, outbreak and shift to co-endemicity of *S. mansoni* is a new and concerning development since *Biomphalaria* species snails were first detected around the southern lakeshores in 2017.^54,56^ Hybrid bovine–human schistosome ova have also been detected in southern Malawi where humans and cattle have regular contact with shared freshwater sources,^57^ while the issue of freshwater faecal contamination in such areas is demonstrated by consistent high-level *E. coli* detection in nearshore waters of Lake Malawi.^58^

The rates of ESBL-producing Enterobacteriaceae gut colonisation are highest in the hospital setting in Malawi, particularly in inpatients receiving antimicrobial treatment,^55^ but community colonisation rates across urban, peri-urban and rural settings remain very high, estimated at >40%.^14^ High colonisation rates of around 30% persist in animal populations, while two-thirds of river water samples tested across southern Malawi contained ESBL-producing Enterobacteriaceae.^14^ The One Health investigations published by Cocker et al.^14^ not only highlight the importance of improved environmental control measures to prevent the spread of ESBLs, but are useful for determining approaches for ongoing surveillance. Of note, human intestinal colonisation with ESBL-producing *E. coli* was associated with advancing age, while colonisation with both resistant *E. coli* and *K. pneumoniae* was strongly associated with the wet season, a reflection of environmental drivers and the role of water contamination in AMR spread.

The increasingly well-described epidemiology of both schistosome and AMR transmission in Malawi has spurred active parallel One Health surveillance. However, comparable epidemiological patterns concerning colonisation and infection of humans and animals with AMR bacteria and schistosomes are also observed elsewhere. Separate studies in Madagascar and Tanzania used phylogenomic methods to analyse whole genome sequences of ESBL-producing *E. coli* isolates collected from human, animal and environmental (water) samples, revealing uniformity across sample types and a high frequency of transmission events between all three groups.^59,60^ Intestinal schistosomiasis is endemic in both countries and therefore opportunities exist to design studies that can maximise sample use and streamline surveillance of multiple pathogens.

In West Africa, interspecific mixing of *S. haematobium* with bovine schistosomes belonging to the *S. haematobium* group, such as *Schistosoma bovis*, is reported across multiple countries.^61^ Senegalese villages with an increased frequency of hybrid human–bovine infections from the *S. haematobium* group are concurrently affected by higher *S. mansoni* infection rates in humans.^62^ The same level of insight linking animal and environmental health systems to AMR and human health are lacking in Senegal, limited to a few studies demonstrating the presence of Enterobacteriaceae harbouring a range of acquired ARGs in human, chimpanzee and chicken faeces.^63–65^ The clinical significance of AMR spread among Enterobacteriaceae was demonstrated in a retrospective analysis at a Senegalese paediatric hospital, where ESBL-production among Enterobacteriaceae isolates causing bloodstream infections during 2012 and 2013 was strongly associated with increased mortality and was confirmed in >50% of cases.^66^ More recent AMR surveillance data from Senegal are sparse, as are surveillance data across many other low-resource settings, despite increasing recognition that AMR poses an urgent and increasing threat to public health.^9^ In addition, One Health approaches have not been upscaled to identify local drivers of AMR colonisation and transmission outside of health facilities, such as freshwater contact, animal husbandry and parasitic co-infection. The case to fund and incentivise interlinked and streamlined co-infection research is strong.

## Implications and recommendations for future research

Our appraisal underscores the importance of taking a rational, interdisciplinary approach to understanding and addressing health issues, particularly in LMICs, where the burden of infectious diseases is highest but resources for disease surveillance are rarely sufficient. Schistosomes can directly cause intestinal pathology, influence microbiota and share similarities with resistant enteric bacteria in terms of environmental transmission, yet relationships between the two remain poorly understood. Collaborative One Health surveillance in SSA has the potential to characterise drivers of AMR in schistosomiasis-endemic communities and to address the following key knowledge gaps: how *Schistosoma* species co-infections and hybrid schistosome infections affect the human intestinal microbiome, whether *S. mansoni* infection and co-infections with other *Schistosoma* species are associated with changes in the faecal resistome and the rates of ESBL-producing Enterobacteriaceae gut colonisation in humans, whether proximity to freshwater or contact with freshwater is associated with AMR colonisation in human and animal intestines and whether freshwater snails act as AMR reservoirs and vectors in aquatic environments. To answer these translational research questions, we recommend the collection of human, animal and environmental samples in lakeshore and riverine communities for testing of multiple pathogens. In our case, these would be parasites and resistant bacteria. However, if suitable specific diagnostic tests exist, there really is no limit to the number of organisms that can be tested for.

An interdisciplinary approach to sampling has the potential to streamline the collection of samples and therefore reduce the cost and effort associated with health surveillance and research initiatives in resource-poor settings, while the analysis of the same samples for the detection of different pathogens that may affect infectivity and/or transmission of others also facilitates the answering of questions surrounding co-infection dynamics, which would otherwise not be possible. Concurrently, with a developing focus on WASH and One Health in AMR control, and the potential of wastewater-based epidemiological studies, collaborative approaches will enable the expansion and transfer of research practices across disciplines, with the collection of WASH and animal contact practice data already being common in parasitological surveys across rural Africa. Coupled with geospatial and demographic data, we propose that such an approach can empower a deeper comprehension of AMR drivers across communities affected by schistosomiasis.

## Conclusions

This review highlights the interconnected environmental factors that influence both schistosomiasis and AMR transmission in LMICs, in addition to examining the evidence surrounding intestinal microbial selection in schistosomiasis and potential impacts on AMR. Knowledge gaps remain, including how *Schistosoma* co-infections affect gut microbiota and whether schistosomiasis is linked to the distribution of key resistance mechanisms in disease-causing bacteria such as ESBL-producing Enterobacteriaceae, both on the individual host level and from the perspective of environmental reservoirs. Through collaborative surveillance initiatives, opportunities exist not only to better understand the drivers of AMR in LMICs, but to link One Health disease control strategies for multiple pathogens and strengthen arguments for improved WASH infrastructure in affected areas to reduce the burden of AMR and parasitic diseases.

## Data Availability

All data cited in this review article are available from the referenced sources.
